# Prevalence and Determinants of Neck and Shoulder Pain Among Saudi Adults: A Cross‐Sectional Study

**DOI:** 10.1155/prm/9087950

**Published:** 2026-06-08

**Authors:** Mohammad A. Jareebi, Yazeed Bader Abutaleb, Sultan Sayil Ghazi Aldalbahi, Adel Ali Alharfi, Faisal Mayudh Althobaiti, Lama Mohammad Barnawi, Noura Mohammed Alateeq, Hajer Othman Alrabea, Ibrahim A. Hakami, Majed A. Ryani, Ahmed A. Bahri, Yahya A. Maslamani, Ali Abdullah Masmali, Abdulwahab A. Aqeeli, Mohammed Alasmari

**Affiliations:** ^1^ Department of Family and Community Medicine, College of Medicine, Jazan University, Jazan, Saudi Arabia, jazanu.edu.sa; ^2^ Department of Surgery, Faculty of Medicine, Jazan University, Jazan, Saudi Arabia, jazanu.edu.sa; ^3^ Department of Surgery, Faculty of Medicine, Shaqra University, Dawadmi, Saudi Arabia, su.edu.sa; ^4^ Department of Surgery, Faculty of Medicine, Tabuk University, Tabuk, Saudi Arabia, ut.edu.sa; ^5^ Department of Surgery, Faculty of Medicine, Almaarefa University, Riyadh, Saudi Arabia, um.edu.sa; ^6^ Department of Orthopedic Surgery, Dawadmi College of Medicine, Shaqra University, Shaqra, Saudi Arabia, su.edu.sa; ^7^ Public Health Authority-Southern Sector, Jazan, Saudi Arabia; ^8^ Radiology Department, King Fahd Central Hospital, Jazan, Saudi Arabia; ^9^ Orthopaedic Surgery Department, Majmaah University, Al Majma’ah, Saudi Arabia, mu.edu.sa

**Keywords:** electronic device use, neck pain, NSP prevalence, risk factors, Saudi Arabia, shoulder pain

## Abstract

**Background:**

Neck and/or shoulder pain (NSP) represents a significant public health burden worldwide. However, nationally representative data from Saudi Arabia remain limited as most existing studies have focused on specific occupational groups or localized populations. This study examines the prevalence, determinants, and risk factors of NSP among Saudi adults.

**Materials and Methods:**

A cross‐sectional study was conducted between May and September 2024 among 2171 adults across Saudi Arabia. Data were collected from participants aged ≥ 18 years using a structured, validated questionnaire assessing NSP prevalence, sociodemographic characteristics, lifestyle factors, and comorbidities.

**Result:**

Among 2171 participants (mean age of 29 ± 12), 54% were females. The overall point prevalence of neck pain was 39.1%, while shoulder pain affected 38.6% of participants. Higher odds of neck pain were associated with use of electronic devices (OR = 1.82, *p* = 0.003), occupational tasks with extended standing (OR = 1.53, *p* = 0.003), and history of trauma (OR = 2.52, *p* < 0.001). Shoulder pain was associated with prolonged use of electronic devices (OR = 2.14, *p* < 0.001) and history of trauma (OR = 4.77, *p* < 0.001). Notably, regular exercise and male sex were protective against both neck pain and shoulder pain compared to females.

**Conclusion:**

NSP is common among Saudi adults and linked to modifiable lifestyle and work‐related factors, underscoring the need for prevention, ergonomic measures, and regular pain management. Long‐term studies are needed to evaluate its impact and treatment outcomes.

## 1. Introduction

Neck and/or shoulder pain (NSP) has emerged as a significant global health burden, ranking as the fourth leading cause of disability worldwide. The Global Burden of Disease Study highlights that NSP contributes to more years lived with disability (YLDs) than conditions, such as diabetes or chronic obstructive pulmonary disease, underscoring its pervasive impact on quality of life (QoL) [1]. In the Middle East and North Africa (MENA) region, neck pain remains a significant public health concern, with an age‐standardized point prevalence of approximately 3066.7 per 100,000 population in 2019 [2]. Gender disparities are also evident, with women reporting a 1.5‐fold higher incidence of chronic NSP compared to men, potentially influenced by hormonal disturbances, occupational exposures, or psychosocial stressors [2, 3].

Beyond demographic differences, several modifiable lifestyle and behavioral factors have been increasingly recognized as important contributors to the development of NSP. Modern lifestyle factors particularly prolonged electronic device usage and sedentary behaviors are key contributors to this growing issue [4, 5]. In addition to these behavioral patterns, environmental and ergonomic factors may also play an important role in the development of NSP. Emerging research implicates poor ergonomic practices in home‐office setups, a trend exacerbated by the postpandemic shift to remote work [6].

As a result of these widespread risk factors, the high prevalence of NSP carries substantial socioeconomic consequences, including reduced work productivity. In 2021, the economic costs of chronic pain in the United States were estimated to be $722.8 billion, including $530.6 billion in medical care costs and $192.2 billion in lost work productivity [7]. In Saudi Arabia, neck pain similarly impacts the workforce, with approximately 16% of workers reporting missed work due to neck pain, highlighting its local economic relevance [8]. Indirect costs, such as presenteeism (reduced productivity while working) and long‐term disability claims, further amplify this burden, particularly in sectors, such as health care, education, and information technology [9]. Despite its impact, clinical management remains predominantly reactive, with 67% of cases seeking care only after symptom chronicity, often resulting in reliance on palliative treatments, such as analgesics, rather than evidence‐based rehabilitation [10].

NSP has been extensively studied in many regions worldwide; however, evidence from Middle Eastern countries, particularly Saudi Arabia, remains comparatively limited. In Saudi Arabia, musculoskeletal disorders account for approximately 20% of primary healthcare visits, yet national estimates of NSP remain insufficiently characterized [11]. The Saudi Vision 2030 initiative emphasizes preventive health care, yet musculoskeletal health remains underrepresented in national wellness campaigns [12]. Recent studies highlight a concerning prevalence of NSP among specific Saudi populations. For instance, 82% of office workers reported neck pain within a 12‐month period, with poor workstation ergonomics and prolonged computer use identified as modifiable risk factors [13]. Similarly, 60.9% of medical students experienced neck pain in the week preceding a study, attributed to prolonged study hours combined with high stress levels [14]. Healthcare professionals are considered a high‐risk group for NSP in Saudi Arabia. The reported prevalence of back, neck, and shoulder pain among the studied surgeons was 68.2%, 56.9%, and 46.2% [15].

While prior research has focused on subgroups, such as healthcare workers and students, broader population‐based studies are needed to develop effective prevention and intervention programs [16]. Additionally, existing studies often lack comprehensive analyses of contributing determinants. For example, cultural factors unique to Saudi Arabia, such as gender‐segregated workplaces, traditional clothing (e.g., heavy head coverings), and driving customs (long commutes in urban areas), may influence NSP prevalence but are rarely investigated [17]. Therefore, this study aims to determine the prevalence of NSP in the Saudi adult population and identify key‐associated determinants. Using a cross‐sectional design with stratified sampling, the study evaluated lifestyle factors, including occupational demands, screen time, physical activity levels, smoking, coffee consumption, and mental health indicators. Findings help to identify its impact on QoL domains and tailor interventions to mitigate NSP’s burden, aligning with national health priorities [18].

## 2. Materials and Methods

This cross‐sectional study was conducted in Saudi Arabia between May and September 2024. The target population comprised adults aged ≥ 18 years who self‐reported NSP irrespective of any duration. Participants were excluded if they had a history of recent trauma (within 6 months), surgery, congenital musculoskeletal disorders, tumors, cardiac conditions that cause referred shoulder/neck pain, or any self‐reported cognitive impairments affecting their ability to complete the questionnaire.

### 2.1. Data Collection Tools

The study utilized a structured, self‐administered electronic questionnaire to collect data from participants across all regions of Saudi Arabia. Participants assessed the survey through a secure link provided with electronic consent. The questionnaire was designed to collect sociodemographic characteristics, such as age, sex, region, residency (urban or rural), education level, income, and employment type. Additionally, it assessed lifestyle factors including physical activity frequency and type, smoking status, coffee consumption, and daily electronic device usage. Health‐related questions covered self‐reported chronic diseases, such as hypertension, diabetes, and depression, as well as any history of neck or shoulder trauma. Pain assessment was conducted through binary (yes/no) questions for NSP, with shoulder pain further categorized by laterality (left or right). To ensure reliability and clarity, the questionnaire underwent pilot testing with 50 participants, demonstrating good internal consistency (Cronbach’s *α* = 0.82), meeting the recommended threshold for research instruments [19]. Missing data were handled by excluding participants with incomplete responses for key variables required for analysis.

### 2.2. Sample Size and Sampling Technique

The study included a total of 2171 adults aged 18 years and older. A stratified random sampling technique was employed to ensure representation across key demographic variables, including geographic region (Central, Eastern, Northern, Southern, and Western), sex, and age groups, in accordance with best practices for population‐based survey research [20]. Sample size calculation was based on an estimated neck pain prevalence of 30%–50% in the general population [21], using a 95% confidence interval and 5% margin of error. Participants were recruited through online platforms, community centers, and healthcare facilities to enhance diversity and minimize selection bias, as recommended in digital and mixed‐mode health surveys [22].

### 2.3. Statistical Analysis

Data analysis was performed using SPSS Version 26. Descriptive statistics (means, standard deviations, frequencies, and percentages) summarized sociodemographic and clinical characteristics. The chi‐square tests and ANOVA were used to compare categorical and continuous variables, respectively, between groups (e.g., neck pain vs. no neck pain). Multivariate logistic regression models were constructed to identify predictors of NSP, with results reported as adjusted odds ratios (ORs) and 95% CI. Statistical significance was set at *p* < 0.05, with Bonferroni correction applied for multiple comparisons.

### 2.4. Ethical Considerations

The study protocol was approved by the Research Ethics Committee at Jazan University (reference number: REC‐45/11/1114, dated 28/05/2024). All participants provided informed consent before completing the questionnaire. Data were anonymized to ensure confidentiality, and participation was voluntary, with the option to withdraw at any time. The study adhered to the principles of the Declaration of Helsinki, and no incentives were offered to participants to avoid coercion.

### 2.5. Use of Generative AI

Generative AI was used solely for language editing. Study design, data, analysis, and interpretation were entirely conducted by the authors.

## 3. Results

### 3.1. Sociodemographic and Lifestyle Characteristics of Participants With NSP

The study included 2171 participants with a mean age of 29 (±12 years), predominantly females (54%) (Table 1). Participants with neck pain were older (30 ± 12 vs. 28 ± 11 years), more likely to be female (63% vs. 48%), reside in the Southern region (19% vs. 15%), and hold postgraduate degrees (7% vs. 5%; *p* < 0.05). They reported more prolonged standing (22% vs. 17%), smart device use > 8 h/day (38% vs. 32%), and lower physical activity (48% vs. 57%). Comorbidities, such as depression (13% vs. 9%) and anxiety (24% vs. 14%), were significantly higher. A history of neck trauma was significantly associated with neck pain (15% vs. 6%) (Table 1).

**TABLE 1:** Sociodemographic and anthropometric characteristics of the study participants (*n* = 2171).

	Neck pain		Shoulder pain	
	Yes	No	Yes	No
Characteristics, mean (SD)				
Age	30 (12)	28 (11)	31.5 (13)	27 (11)
Height	164 (9.1)	166 (9.5)	163.5 (9.3)	166 (9.4)
Weight	68 (19)	69 (19)	69 (19.5)	69 (19)
BMI	25 (6.6)	25 (5.7)	25.5 (6.3)	25 (5.8)
Characteristics, frequency (%)				
Sex				
Male	37%	52%	37.5%	51%
Female	63%	48%	62.5%	49%
Region				
Central region	26%	28%	28%	27%
Eastern region	21%	26%	19.5%	26%
Northern region	15%	13%	14.5%	14%
Southern region	19%	15%	19%	15%
Western region	19%	18%	19.5%	18%
Residency				
City	82%	84%	81%	84%
Village	18%	16%	19%	16%
Education level				
Secondary school and less	19%	23%	20.5%	21%
University (bachelor’s and diploma)	74%	72%	71.5%	73%
Postgraduate studies (master’s, PhD, and others)	7%	5%	8%	5%
Employment				
Engaging in tasks that involve frequent manual handling of heavy loads	2%	2%	1.5%	2%
Performing sedentary work requiring prolonged periods of sitting	43%	43%	43%	43%
Undertaking work that necessitates extended periods of standing	22%	17%	20.5%	18%
Income/month				
Less than 5000	57%	62%	55%	63%
5000–9999	19%	17%	20%	17%
10,000–14,999	13%	10%	13.5%	9%
≥ 15,000	11%	11%	12%	11%
Smart device use				
2 h or less	7%	8%	6.5%	8%
2–4 h	20%	17%	21.5%	16%
4–8 h	35%	43%	35.5%	42%
8 h or more	38%	32%	36.5%	34%
Smoking				
Ex‐smoker	4%	6%	5%	5%
Never	81%	82%	82%	81%
Current	15%	13%	13.5%	14%
Coffee consumption	85%	86%	85%	86%
Coffee consumption/weak				
Less than three times a week	25%	27%	25.5%	27%
Three times a week and more	60%	59%	60%	60%
Exercise	48%	57%	46.5%	57%
Exercise/week				
Once a week	20%	17%	19%	18%
Two to three times	24%	24%	22.5%	25%
Comorbidities				
Hypertension	6%	4%	6.5%	4%
Diabetes	10%	8%	11%	7%
Heart disease	2%	1%	2%	1%
Blood disease	2%	1%	2.5%	1%
Asthma and COPD	10%	5%	10%	5%
Osteoporosis	2%	1%	1.5%	1%
Cancer	1%	0%	0.5%	0%
Depression	13%	9%	11%	9%
Anxiety	24%	14%	22.5%	15%
Musculoskeletal‐related characteristics				
Neck trauma history	15%	6%	—	—
Shoulder trauma history	—	—	22.5%	7%

Shoulder pain participants were of older age (31.5 ± 13 vs. 27 ± 11 years), female gender (62.5% vs. 49%), and had higher postgraduate education (8% vs. 5%), with lower representation from the Eastern region (19.5% vs. 26%). Affected individuals exercised less (46.5% vs. 57%), used smart devices more (21.5% used 2–4 h/day vs. 16%), and had higher rates of depression (11% vs. 9%) and anxiety (22.5% vs. 15%). Shoulder trauma was common (22.5% vs. 7%) (Table 1).

### 3.2. Prevalence of NSP

Regarding NSP prevalence, 39.1% (*n* = 850) reported neck pain, while 38.6% (*n* = 839) reported shoulder pain. Shoulder pain showed laterality differences, affecting the left shoulder in 16.4% (*n* = 356) and the right shoulder in 22.2% (*n* = 483).

### 3.3. Determinants of NSP

Multiple logistic regression analyses were conducted to identify factors associated with NSP among Saudi adults (Table 2). Age was identified as a significant factor for both neck (OR = 1.02) and shoulder pain (OR = 1.04). Gender differences revealed that males had significantly lower odds of experiencing both neck (OR = 0.55) and shoulder pain (OR = 0.50) compared to females. Individuals with a history of neck trauma had a notably higher OR of 2.52, and those with a history of shoulder trauma had an even higher OR of 4.77, underscoring its strong association with NSP. Additionally, the use of electronic devices was significantly associated with both shoulder and neck pain. For shoulder pain, participants using devices for 2–4 h (OR = 2.08), 4–8 h (OR = 1.71), and more than 8 h daily (OR = 2.14) had notably higher odds of experiencing discomfort. Similarly, for neck pain, device usage for 2–4 h (OR = 1.67) and over 8 h per day (OR = 1.82) was related to a remarkably higher risk. Engaging in regular exercise was associated with significantly lower odds of both neck pain (OR = 0.81) and shoulder pain (OR = 0.71), indicating a protective effect against musculoskeletal discomfort.

**TABLE 2:** Association of study variables with NSP.

Predictors	Neck pain			Shoulder pain		
	OR	95% CI	*p* value	OR	95% CI	*p* value
Age	1.02	1.01–1.03	**0.001**	1.04	1.02–1.05	**< 0.001**
Gender (reference: female)						
Male	0.55	0.41–0.74	**< 0.001**	0.5	0.39–0.62	**< 0.001**
Region (reference: central region)						
Eastern region	1.03	0.78–1.35	0.844	0.79	0.60–1.04	0.098
Northern region	1.41	1.05–1.90	**0.024**	1.05	0.77–1.42	0.764
Southern region	1.62	1.18–2.23	**0.003**	1.2	0.87–1.65	0.26
Western region	1.08	0.81–1.43	0.605	0.93	0.70–1.24	0.638
Residence (reference: urban)						
Rural	1.07	0.81–1.40	0.654	1.28	0.97–1.69	0.082
Education (reference: high school or lower)						
Bachelor	1.18	0.93–1.50	0.166	0.97	0.77–1.24	0.827
Postgraduates	1.24	0.79–1.96	0.35	1.05	0.66–1.66	0.832
Employment (reference: unemployed)						
Engaging in tasks that involve frequent manual handling of heavy loads	1.8	0.87–3.69	0.11	1.24	0.57–2.60	0.574
Performing sedentary work requiring prolonged periods of sitting	1.18	0.95–1.48	0.142	1.15	0.91–1.44	0.241
Undertaking work that necessitates extended periods of standing	1.53	1.16–2.02	**0.003**	1.22	0.92–1.61	0.174
Income (reference: less than 5000 SR)						
Between 5000–9999 SR	0.95	0.72–1.25	0.718	1.05	0.79–1.38	0.742
Between 10,000–15,000 SR	1.02	0.72–1.44	0.909	1.07	0.76–1.52	0.691
> 15,000 SR	0.87	0.60–1.25	0.466	0.76	0.53–1.11	0.156
Use of electronic devices (reference: less than 2 h)						
2–4 h	1.67	1.11–2.52	**0.014**	2.08	1.37–3.16	**0.001**
4–8 h	1.37	0.93–2.04	0.113	1.71	1.15–2.57	**0.008**
> 8 h	1.82	1.22–2.74	**0.003**	2.14	1.42–3.25	**< 0.001**
BMI	1.11	0.97–1.27	0.133	1.01	0.99–1.03	0.228
Exercise	0.81	0.67–0.99	**0.036**	0.71	0.58–0.86	**< 0.001**
Smoking history (reference: never)						
Ex‐smoker	0.93	0.57–1.49	0.771	1.35	0.85–2.12	0.197
Current	1.68	1.24–2.28	**0.001**	1.17	0.86–1.58	0.321
History of trauma (reference: no)						
Yes	2.52	1.84–3.46	**< 0.001**	4.77	3.59–6.39	**< 0.001**

*Note:* Bold values indicate statistical significance.

Engaging in work requiring prolonged standing was considerably linked with neck pain (OR = 1.53). Geographical disparities also revealed significant associations with neck pain, where participants from the Northern region have an OR of 1.41, while those from the Southern region had an even higher OR of 1.62, compared to the Central region. Additionally, current smokers showed an OR of 1.68 for neck pain, indicating a significant link between smoking and NSP prevalence.

## 4. Discussion

This cross‐sectional study revealed a high prevalence of NSP among Saudi adults, with 39.1% reporting neck pain and 38.6% reporting shoulder pain. Male sex, older age, physical inactivity, and prolonged electronic device use were all associated with higher NSP prevalence. Multivariate analysis identified several significant predictors, including age, trauma history, occupational standing, and excessive device use, while regular physical activity demonstrated a modest protective effect.

The observed prevalence rates in this study align with global data, where NSP ranges from 30% to 50%, according to the Global Burden of Disease studies [23]. Our findings also mirror a pooled global prevalence of 37% for neck pain reported across 21 countries [24], and the reported incidence of shoulder pain ranged from 7.7 to 62/1000 persons/year in a recent systematic review [25]. These consistencies support the growing recognition of NSP as a major public health concern globally. Regionally, the prevalence rates reported in this study are higher than those documented in neighboring Gulf countries, such as the UAE (62% for neck pain) [26] and Kuwait (21% for neck pain, 13% for shoulder pain) [27]. This disparity may be attributable to several factors unique to Saudi Arabia, including widespread smartphone usage (65.18%) [28], a high rate of sedentary behavior (58% of adults are insufficiently active) [11], and an increasing shift toward white‐collar employment [29]. These elements collectively foster musculoskeletal strain through poor posture and reduced physical activity. Another contributing factor could be ergonomic unawareness in both domestic and occupational environments, where prolonged device use is common without appropriate support [30]. Conversely, some regional studies have reported lower NSP rates (20%–25%) [31], possibly reflecting differences in study populations, sampling techniques, or occupational exposures, as higher NSP rates (50%–60%) have been reported among manual laborers in low‐income countries [32], likely due to repetitive physical strain, poor working conditions, and heavy lifting [33]. Therefore, occupational profile and ergonomic environment appear to significantly mediate NSP prevalence.

This study showed that males are less affected by NSP compared to females. This paradox reflects: (1) behavioral confounders—Saudi women average 8 h daily on smartphone compared to men (8 vs 7 h, *p* < 0.001) [34]; (2) healthcare‐seeking behavior—women are 2.3× more likely to report pain (MOH, 2022) [35]. Global data confirm this pattern (crude OR = 1.4 reduces to 1.1 after adjustment), emphasizing the need for multifactorial risk assessment [36]. However, Scandinavian research reported persistent female risk (OR = 1.3), likely due to equitable workplace policies minimizing gender‐specific exposures [33].

Our analysis demonstrated a dose‐dependent relationship between electronic device use and NSP. A significantly higher prevalence of shoulder pain (50%) was observed among individuals with high smartphone use, demonstrating a dose‐dependent association (*p* < 0.05) [37]. These findings align with a biomechanical model, which shows that 60° neck flexion during device use amplifies cervical spine load to 27 kg compared to 5 kg in a neutral position [30]. However, study findings of a Japanese teacher’s cohort study, which reported Internet users on weekdays, had almost double the odds of neck pain compared to nonusers [38]. This disparity may reflect cultural and environmental differences. These differences underscore the need to contextualize NSP risk factors within local usage and occupational habits.

The study highlighted the significant association of regular exercise with NSP, with physically active participants showing 9% lower pain prevalence. These findings align with meta‐analysis of 42 workplace intervention studies (pooled OR = 0.78, 95% CI 0.71–0.85) [39], and regional data from Ethiopia showed only 11.5% of participants achieved the WHO‐recommended physical activity threshold, supporting evidence from that regular exercise offers a protective effect against musculoskeletal disorders [40]. Recent evidence from a 2024 network meta‐analysis indicated that shoulder‐specific exercise therapy is effective in reducing chronic shoulder pain [41]. This may be attributed to enhanced muscular endurance, which reduces postural fatigue.

To avoid capturing acute episodes that may not reflect chronic NSP, participants with trauma within the last 6 months were excluded. However, prior trauma, occurring more than 6 months ago, demonstrated strong associations with NSP. Specifically, previous neck injury increased the odds of neck pain. Pooled analysis of the six studies indicated an unadjusted relative risk of future NP in the vehicle trauma‐exposed population with neck injury of 2.3 (95% CI [1.8, 3.1]), which equates to a 57% attributable risk under the exposed [42]. These pronounced effects likely stem from multiple systemic factors: Rehabilitation gaps remain evident in Saudi Arabia, where access to and adherence with physiotherapy services are suboptimal. Saudi orthopedic patients show only ∼56% attendance in regular physiotherapy sessions [43]. The findings highlight critical opportunities to strengthen postinjury care through Saudi Vision 2030’s health reforms, particularly by expanding rehabilitation access and reducing barriers to early intervention.

The study found prolonged occupational standing may enhance the neck pain risk by 53% (OR = 1.53, 95% CI 1.16–2.02), aligning with previous research on workplace ergonomics. These results corroborate findings from a 2022 Saudi healthcare worker study showing 47% higher neck pain prevalence among staff with > 4 h daily standing [36], and global data from office workers demonstrating 1.6× increased risk with sustained upright postures [33]. The mechanism likely involves: static cervical spine loading increasing disk pressure by 35%–40% compared to dynamic movements [41].

Our study identified significant associations between chronic conditions and NSP, with depression and anxiety showing particularly strong relationships, supporting Saudi biopsychosocial model demonstrating shared neural pathways between mood disorders and pain processing [36]. However, our effect sizes exceed those reported in European cohorts (depression OR = 1.2, asthma OR = 1.4) [44], potentially reflecting: (1) higher baseline inflammation in Saudi populations [45]; (2) greater mental health stigma delaying treatment (only 28% of depressed Saudis seek care vs 45% in the EU). These findings highlight the need for integrated chronic disease–pain management approaches in Saudi clinical practice.

Given the high prevalence and multifactorial nature of NSP, the findings of this study carry important implications for public health, particularly in their potential impact on QoL domains. Proactive measures could include workplace ergonomic assessments, community‐based physical activity initiatives, and integration of musculoskeletal health screenings into primary care [46]. Additionally, leveraging digital health tools (e.g., telehealth physiotherapy and posture‐tracking apps) could bridge gaps in access to care, particularly in rural regions [47]. Future research should focus on evaluating the specific QoL impairments associated with NSP in this population and assess the effectiveness of targeted interventions aimed at mitigating its impact.

Several limitations of this study should be acknowledged. First, the self‐administered survey relied on participants’ recall, which may introduce recall bias, particularly for past trauma and cognitive impairment. Similarly, the use of self‐reported measures for NSP could result in misclassification or reporting bias, potentially underestimating or overestimating true prevalence. The cross‐sectional design limits causal inference, as temporality between exposures (e.g., electronic device use and physical activity) and outcomes cannot be established. The online survey design may limit generalizability to digitally excluded populations, and residual confounding from unmeasured variables, such as ergonomic conditions, could persist despite multivariate adjustments. Binary questions were used to assess NSP to facilitate efficient data collection and clear case classification in this large‐scale epidemiological study. While this approach is appropriate for estimating prevalence and identifying associated factors, it does not capture pain severity; thus, future research may incorporate validated pain scales for more comprehensive assessment.

To control NSP issue, strategies should focus on limiting prolonged device use, implementing ergonomic training, and encouraging regular physical activity, as these factors were identified as significant determinants of pain in this population. Adoption of such interventions in workplaces, schools, and community health programs may reduce the burden of chronic neck and shoulder pain.

## 5. Conclusion

This study reveals that more than one‐third of Saudi adults experience NSP, highlighting NSP as a significant public health concern. These prevalent conditions are influenced by multiple factors including modifiable lifestyle factors, such as prolonged device use, physical inactivity, and occupational postures, alongside nonmodifiable factors, such as age and trauma history. The research benefits from robust methodology featuring its large national sample and comprehensive assessment of biopsychosocial risk factors. Future research should employ longitudinal designs with clinical assessments to establish causality and evaluate interventions targeting high‐risk groups, particularly in workplace settings.

## Author Contributions

Mohammad A. Jareebi conceptualized and supervised the study, contributed to study design, data interpretation, and critical revision of the manuscript. Yazeed Bader Abutaleb, Sultan Sayil Ghazi Aldalbahi, and Adel Ali Alharfi were involved in data collection, entry, and initial analysis. Faisal Mayudh Althobaiti, Lama Mohammad Barnawi, Noura Mohammed Alateeq, and Hajer Othman Alrabea contributed to the literature review, questionnaire distribution, and data management. Ibrahim A. Hakami, Majed A. Ryani, and Ahmed A. Bahri performed statistical analysis and assisted in writing the results section. Yahya A. Maslamani and Ali Abdullah Masmali provided expert review, contributed to interpretation of findings, and revised the manuscript for intellectual content. Abdulwahab A. Aqeeli and Mohammed Alasmari supported final editing and formatting of the manuscript.

## Funding

This research did not receive any specific grant from funding agencies in the public, commercial, or not‐for‐profit sectors.

## Disclosure

No other individuals or third‐party services were involved in the research process or the preparation of this manuscript. All authors read and approved the final version of the manuscript.

## Ethics Statement

This study was approved by the Jazan University’s Scientific Research Ethics Committee (reference number: REC‐45/11/1114, dated 28/05/2024).

## Consent

Participants provided digital informed consent.

## Conflicts of Interest

The authors declare no conflicts of interest.

## Data Availability

The datasets used and/or analyzed during the current study are available from the corresponding author upon reasonable request.

## References

[1] Ahangar-Sirous R. , Alizadeh M. , Nejadghaderi S. A. et al., The Burden of Neck Pain in the Middle East and North Africa Region, 1990–2019, Heliyon. (2023) 9, no. 11, 10.1016/j.heliyon.2023.e21296. PMC10643100 38027849

[2] Chen X. , O’Leary S. , and Johnston V. , Gender Differences in Prevalence and Risk Factors for Neck Pain in Office Workers: a Systematic Review, Journal of Occupational Rehabilitation. (2021) 31, no. 2, 366–379.

[3] Xie Y. , Szeto G. , Dai J. , and Madeleine P. , A Comparison of Muscle Activity in Using Touchscreen Smartphone Among Young People with and Without Chronic neck-shoulder Pain, Ergonomics. (2016) 59, no. 1, 61–72, 10.1080/00140139.2015.1056237. 26218600

[4] Al-Hazzaa H. M. , Physical Inactivity in Saudi Arabia Revisited: a Systematic Review of Inactivity Prevalence and Perceived Barriers to Active Living, International Journal of Health Sciences. (2018) 12, no. 6, 50–64. PMC6257875 30534044

[5] O′Donoghue G. , Perchoux C. , Mensah K. et al., A Systematic Review of Correlates of Sedentary Behaviour in Adults Aged 18-65 Years: a socio-ecological Approach, BMC Public Health. (2016) 16, no. 1, 10.1186/s12889-016-2841-3. PMC4756464 26887323

[6] Guy Jr G. P. , Miller G. F. , Legha J. K. et al., Economic Costs of Chronic pain—United States, 2021, Medical Care. (2025) 63, no. 9, 679–685, 10.1097/MLR.0000000000002181. 40730349 PMC13284907

[7] Alhazmi M. H. , Khired Z. , Alhazmi M. A. et al., The Impact of Neck Pain on Work Productivity and Quality of Life Among Office Workers in Jazan: a Cross-Sectional Study, Cureus. (2024) 16, no. 12, 10.7759/cureus.76254. PMC11752947 39845229

[8] Stewart W. F. , Ricci J. A. , Chee E. , Morganstein D. , and Lipton R. , Lost Productive Time and Cost due to Common Pain Conditions in the US Workforce, JAMA. (2003) 290, no. 18, 2443–2454, 10.1001/jama.290.18.2443. 14612481

[9] Binder A. I. , Neck Pain, BMJ Clin Evid. (2008) 2008. PMC2907992 19445809

[10] Al-Arfaj A. S. , Al-Saleh S. S. , Alballa S. R. et al., How Common Is Back Pain in Al-Qaseem?, Saudi Medical Journal. (2003) 24, no. 2, 170–173, 10.15537/1658-3175.1963. 12682682

[11] Saudi Vision 2030 , Health Sector Transformation Program, 2021, https://www.vision2030.gov.sa.

[12] Johnston V. , Consequences and Management of Neck Pain by Female Office Workers: Results of a Survey and Clinical Assessment, Arch Physiother. (2016) 6, no. 1, 10.1186/s40945-016-0023-3. PMC5759915 29340190

[13] Alshagga M. A. , Nimer A. R. , Yan L. P. , Ibrahim I. A. A. , Al-Ghamdi S. S. , and Radman Al-Dubai S. A. , Prevalence and Factors Associated with Neck, Shoulder and Low Back Pains Among Medical Students in a Malaysian Medical College, BMC Research Notes. (2013) 6, no. 1, 10.1186/1756-0500-6-244. PMC3733931 23815853

[14] Alshareef L. , Al Luhaybi F. , Alsamli R. S. et al., Prevalence of Back and Neck Pain Among Surgeons Regardless of Their Specialties in Saudi Arabia, Cureus. (2023) 15, no. 11, 10.7759/cureus.49421. PMC10750446 38149136

[15] Almogbil I. H. , Alrashidi L. R. , Alhajlah R. S. et al., Prevalence of Shoulder and Neck Pain Among Healthcare Workers in the Central Region of Saudi Arabia, Cureus. (2023) 15, no. 7, 10.7759/cureus.42286. PMC10440718 37609082

[16] Alghadir A. , Zafar H. , Iqbal Z. A. , and Al-Eisa E. , Work-Related Low Back Pain Among Physical Therapists in Riyadh, Saudi Arabia, Workplace Health & Safety. (2017) 65, no. 7, 337–345, 10.1177/2165079916670167. 28121518

[17] AlOmar R. S. , AlShamlan N. A. , Alawashiz S. , Badawood Y. , Ghwoidi B. A. , and Abugad H. , Musculoskeletal Symptoms and Their Associated Risk Factors Among Saudi Office Workers: a cross-sectional Study, BMC Musculoskeletal Disorders. (2021) 22, no. 1, 10.1186/s12891-021-04652-4. PMC8422646 34488714

[18] Tavakol M. and Dennick R. , Making Sense of Cronbach’s Alpha, International Journal of Medical Education. (2011) 2, 53–55, 10.5116/ijme.4dfb.8dfd. 28029643 PMC4205511

[19] Lohr S. L. , Sampling: Design and Analysis, 2021, 2nd edition, CRC Press.

[20] Hoy D. , March L. , Woolf A. et al., The Global Burden of Neck Pain: Estimates from the Global Burden of Disease 2010 Study, Annals of the Rheumatic Diseases. (2014) 73, no. 7, 1309–1315, 10.1136/annrheumdis-2013-204431. 24482302

[21] Dillman D. A. , Smyth J. D. , and Christian L. M. , Internet, Phone, Mail, and mixed-mode Surveys: The Tailored Design Method, 2014, 4th edition, Wiley.

[22] GBD 2019 Diseases and Injuries Collaborators , Global Burden of 369 Diseases and Injuries in 204 Countries and Territories, 1990-2019: a Systematic Analysis for the Global Burden of Disease Study 2019, Lancet. (2020) 396, no. 10258, 1204–1222, 10.1016/S0140-6736(20)30925-9. 33069326 PMC7567026

[23] Hoy D. , March L. , Woolf A. et al., The Global Burden of Neck Pain: Estimates from the Global Burden of Disease 2010 Study, Annals of the Rheumatic Diseases. (2014) 73, no. 7, 1309–1315, 10.1136/annrheumdis-2013-204431. 24482302

[24] Lucas J. , Van Doorn P. , Hegedus E. , Lewis J. , and Van Der Windt D. , A Systematic Review of the Global Prevalence and Incidence of Shoulder Pain, BMC Musculoskeletal Disorders. (2022) 23, no. 1, 10.1186/s12891-022-05973-8. PMC9730650 36476476

[25] Mohamed A. M. , Abbara M. A. , Bashier S. A. , Elkhidir D. A. , Hussein A. , and Ranade A. V. , Neck Pain and Distance Learning: a Pain in the Neck for University Students During COVID-19, F1000Research. (2024) 13, 10.12688/f1000research.145874.2. PMC11809631 39931325

[26] Alrowayeh H. N. , Alshatti T. A. , Aljadi S. H. , Fares M. , Alshamire M. M. , and Alwazan S. S. , Prevalence, Characteristics, and Impacts of work-related Musculoskeletal Disorders: a Survey Among Physical Therapists in the State of Kuwait, BMC Musculoskeletal Disorders. (2010) 11, no. 1, 10.1186/1471-2474-11-116. PMC2905326 20540724

[27] Alotaibi M. S. , Fox M. , Coman R. , Ratan Z. A. , and Hosseinzadeh H. , Smartphone Addiction Prevalence and Its Association on Academic Performance, Physical Health, and Mental Well-Being Among University Students in Umm Al-Qura University (UQU), Saudi Arabia, International Journal of Environmental Research and Public Health. (2022) 19, no. 6, 10.3390/ijerph19063710. PMC8954621 35329397

[28] Alhowimel A. , Alotaibi M. , Alqahtani M. , and Alshehri F. , Psychosocial Predictors of Pain and Disability Outcomes in People with Chronic Low Back Pain, Physiotherapy Theory and Practice. (2023) 39, no. 1, 167–178, 10.2147/JMDH.S343494.

[29] Hansraj K. K. , Assessment of Stresses in the Cervical Spine Caused by Posture and Position of the Head, Surgical Technology International. (2014) 25, 277–279. 25393825

[30] Andersen L. L. , Fallentin N. , Thorsen S. V. , and Holtermann A. , Physical Workload and Risk of long-term Sickness Absence in the General Working Population and Among blue-collar Workers: Prospective Cohort Study with Register follow-up, Occupational and Environmental Medicine. (2016) 73, no. 4, 246–253, 10.1136/oemed-2015-103314. 26740688

[31] Punnett L. and Wegman D. H. , Work-Related Musculoskeletal Disorders: the Epidemiologic Evidence and the Debate, Journal of Electromyography and Kinesiology. (2004) 14, no. 1, 13–23, 10.1016/j.jelekin.2003.09.015. 14759746

[32] Geneen L. J. , Martin D. J. , Adams N. et al., Effects of Education to Facilitate Knowledge About Chronic Pain for Adults: a Systematic Review with meta-analysis, Systematic Reviews. (2015) 4, no. 1, 10.1186/s13643-015-0120-5. PMC4591560 26428467

[33] Begum F. , Sayed S. A. M. , Almalki M. , Ahmed R. M. , and El-slamoni M. A. A. , Time Spent on Digital Screen and Its Impact on Health and Academic Performance of Youth, Saudi Journal of Nursing and Health Care. (2022) 5, no. 8, 161–166, 10.36348/sjnhc.2022.v05i08.001.

[34] Saudi Ministry of Health , Annual Health Statistics Report 2022, 2022, MOH.

[35] Côté P. , Wong J. J. , Sutton D. et al., Management of Neck Pain and Associated Disorders: a Clinical Practice Guideline from the Ontario Protocol for Traffic Injury Management (Optima) Collaboration, European Spine Journal. (2023) 32, no. 4, 1057–1089, 10.1007/s00586-016-4467-7. 26984876

[36] Deivendran G. , Kanagaraj T. S. , Leelabai B. S. et al., Association Between Screen Time and Musculoskeletal Pain Among Young Adults: a Cross-Sectional Study from SRM Smedical College, Tamil Nadu, Journal of Pharmacy and BioAllied Sciences. (2025) 17, no. 2, 87–89, 10.4103/jpbs.jpbs_943_25. 40860003 PMC12373370

[37] Tanabe R. , Hisamatsu T. , Fukuda M. et al., The Association Between Problematic Internet Use and Neck Pain Among Japanese Schoolteachers, Journal of Occupational Health. (2021) 63, no. 1, 10.1002/1348-9585.12298. PMC8661443 34888997

[38] Shariat A. , Cleland J. A. , Danaee M. , Kargarfard M. , Sangelaji B. , and Tamrin S. B. , Effects of Stretching Exercise Training and Ergonomic Modifications on Musculoskeletal Discomforts of Office Workers: a Randomized Controlled Trial, International Journal of Occupational Safety and Ergonomics. (2023) 29, no. 1, 10–19, 10.1016/j.bjpt.2017.09.003. PMC5883995 28939263

[39] Temesgen M. H. , Belay G. J. , Gelaw A. Y. , Janakiraman B. , and Animut Y. , Burden of Shoulder and/neck Pain Among School Teachers in Ethiopia, BMC Musculoskeletal Disorders. (2019) 20, no. 1, 10.1186/s12891-019-2397-3. PMC6329165 30630454

[40] Silveira A. , Lima C. , Beaupre L. , Chepeha J. , and Jones A. , Shoulder Specific Exercise Therapy Is Effective in Reducing Chronic Shoulder Pain: a Network meta-analysis, PLoS One. (2024) 19, no. 4, 10.1371/journal.pone.0294014. PMC11057978 38683828

[41] Nolet P. S. , Emary P. C. , Kristman V. L. , Murnaghan K. , Zeegers M. P. , and Freeman M. D. , Exposure to a Motor Vehicle Collision and the Risk of Future Neck Pain: a Systematic Review and Meta-Analysis, Pharmacy Management R. (2019) 11, no. 11, 1228–1239, 10.1002/pmrj.12173. PMC6899867 31020768

[42] Alzakri A. A. , Alsultan O. A. , Alhuqbani M. N. et al., Barriers and Facilitators to Physiotherapy Among Adult Orthopedic Patients at King Khalid University Hospital, Riyadh, Kingdom of Saudi Arabia: a cross-sectional Study, Saudi Medical Journal. (2023) 44, no. 7, 679–686, 10.15537/smj.2023.44.7.20230276. 37463715 PMC10370374

[43] Smith B. H. , Elliott A. M. , Hannaford P. C. , Chambers W. A. , Smith W. C. , and Penny K. , The Impact of Chronic Pain in the Community, Family Practice. (2001) 18, no. 3, 292–299, 10.1093/fampra/18.3.292. 11356737

[44] Habib S. S. , Level of high-sensitivity C-reactive Protein in Saudi Patients with Chronic Stable Coronary Artery Disease, Journal of Ayub Medical College, Abbottabad. (2008) 20, no. 2, 3–6. 19385446

[45] Ris I. , Søgaard K. , Gram B. , Agerbo K. , Boyle E. , and Juul-Kristensen B. , Does a Combination of Physical Training, Specific Exercises and Pain Education Improve health-related Quality of Life in Patients with Chronic Neck Pain? A Randomised Control Trial with a 4-month Follow up, Manual Therapy. (2016) 26, 132–140, 10.1016/j.math.2016.08.004. 27598552

[46] Valentijn P. P. , Tymchenko L. , Jacobson T. et al., Digital Health Interventions for Musculoskeletal Pain Conditions: Systematic Review and meta-analysis of Randomized Controlled Trials, Journal of Medical Internet Research. (2022) 24, no. 9, 10.2196/37869. PMC9490534 36066943

[47] Bd 2017 Dalys and Collaborators H. A. L. E. , Global, Regional, and National disability-adjusted life-years (Dalys) for 359 Diseases and Injuries and Healthy Life Expectancy (HALE) for 195 Countries and Territories, 1990-2017: a Systematic Analysis for the Global Burden of Disease Study 2017, Lancet. (2018) 392, no. 10159, 1859–1922, 10.1016/S0140-6736(18)32335-3. 30415748 PMC6252083

